# Acute Intermittent Porphyria Attack Triggered by COVID-19 Infection

**DOI:** 10.7759/cureus.37412

**Published:** 2023-04-10

**Authors:** Ali Emre Bardak, Mehmet Oğuzcan Alada, Naci Senkal, Alpay Alibeyoğlu, Murat Köse

**Affiliations:** 1 Internal Medicine, Istanbul University School of Medicine, Istanbul, TUR

**Keywords:** rhabdomyolysis, heme metabolism, hemin, acute intermittent porphyria, porphyria, covid-19

## Abstract

A 45-year-old male patient who was diagnosed with acute intermittent porphyria (AIP) four years ago and had his last episode two years prior presented to our clinic with an AIP attack complicated with rhabdomyolysis triggered by coronavirus disease 2019 (COVID-19) infection. Although there are well-known factors that might trigger an AIP attack, some studies also showed an association of COVID-19 with porphyria. These studies suggest that the accumulation of by-products in the heme synthesis pathway during COVID-19 infection may cause attacks mimicking acute intermittent porphyria. In respect to that, in the early phases of the pandemic, hypotheses emerged arguing the treatment of severe COVID-19 infections with hemin as the treatment of an AIP attack. In our instance, after a two-year period during which there had not been an episode, there was no evident cause other than COVID-19 infection. We believe that patients with porphyria are particularly prone to exacerbations during a COVID-19 infection and should be monitored carefully.

## Introduction

Porphyria refers to a class of disorders caused by the deficiency or defects in the enzymes in the heme synthesis pathway resulting in the accumulation of by-products. Acute intermittent porphyria (AIP) is an autosomal dominant disorder with varying penetrance belonging to this class and related to the deficiency of porphobilinogen deaminase and accumulation of porphobilinogen (PBG) and delta-aminolevulinic acid (ALA) [1]. AIP manifests with recurrent attacks with neurologic and visceral symptoms including abdominal pain, gastrointestinal dysfunction, muscle weakness, sensory and motor deficits, convulsion, mood disorders, etc., that are attributable to accumulated PBG and ALA [2]. A multitude of factors, such as medications, infections, stress, alcohol, fasting, and hormonal alterations can provoke an AIP attack. AIP has also closely been linked to systemic inflammation [3].

In the early stages of the severe acute respiratory syndrome coronavirus 2 (SARS-CoV-2) coronavirus disease 2019 (COVID-19) pandemic, before the vaccination process in particular, an extensive investigation was conducted to comprehend the disease's nature in the search for effective treatment methods. San Juan et al. reported elevated levels of uroporphyrin I, coproporphyrins I and III [4], and Pang et al. reported that heme biosynthesis was one of the most profoundly disturbed pathways in COVID-19 [5]. These studies documented the interference of COVID-19 infection in heme biosynthesis.

Hemin, which is synthetic heme, inhibits ALA synthetase (ALAS) and is used for the treatment of AIP attacks. Hemin is also known to induce heme oxygenase-1 (HO-1) which was reported to have anti-inflammatory properties [6]. Thus, hemin induction of HO-1 was suggested as a potential therapeutic strategy against systemic inflammation in COVID-19 infection [7].

In our case report, we aimed to explain the relationship between COVID-19 and AIP based on these features.

## Case presentation

A 45-year-old male patient with a history of AIP and unvaccinated for COVID-19 presented to our clinic with complaints of abdominal pain, nausea, generalized muscle pain, dark urine, and low-grade fever (37.9 °C). He had his last episode of AIP two years ago and had been latent since then. It was discovered that he had been exposed to COVID-19 infection by his colleague. The COVID-19 polymerase chain reaction (PCR) test was positive. At the time of the COVID-19 diagnosis, he had no respiratory symptoms. Lab workup revealed neutrophilic leukocytosis (WBC:15600/µL, Neutrophils:13700/µL), lymphopenia (Lymph:700/µL), lactate dehydrogenase (LDH) 358 IU/L, aspartate aminotransferase (AST) 223 IU/L, alanine aminotransferase (ALT) 62 IU/L, gamma-glutamyl transferase (GGT) 26 IU/L, alkaline phosphatase (ALP) 98 IU/L and C-reactive protein (CRP) 31 mg/L. A qualitative urine porphobilinogen test resulted positive that was confirmative of an AIP attack. AIP attack exacerbating factors were evaluated for the patient. He was not a smoker and did not use recreational drugs or consume alcohol for at least a week. None of his medications were known to be triggers. Anti-hepatitis A virus (HAV) immunoglobulin M (IgM), anti-hepatitis B core (HBc) IgM, hepatitis B surface antigen (HBs Ag), and anti-hepatitis C virus (HCV) were all negative.

The treatment was started with dextrose-containing solutions and intravenous hemin (4 mg/kg/day). On the second day of the treatment, liver enzymes and LDH peaked, AST at 1862 IU/L, ALT at 579 IU/L, and LDH at 2190 IU/L. Creatine kinase (CK) was 113.900 IU/L. In the meantime, the patient's muscle pain was aggravated. Urinalysis revealed 3+ erythrocyte and 14 erythrocyte cells. It was evaluated as myoglobinuria. The condition was attributed to rhabdomyolysis. Rhabdomyolysis was considered to be secondary to AIP. The AIP attack and the toxic effects of hemin on the liver were thought to be contributing factors alongside rhabdomyolysis to the elevated liver enzyme pattern (Table 1). The international normalized ratio (INR) values remained within the normal range throughout the treatment. The patient developed dyspnea on the third day, his oxygen saturation was consistently higher than 95% on arterial blood gas and finger tip pulse oximeter. Non-enhanced thorax computed tomography (CT) scan revealed bilateral ground-glass opacities. He was started on daily 40 mg intravenous methylprednisolone treatment. On the fourth day, his oxygen saturation dropped to 88% to %90 in room air and was higher than 95% under 2-3 L/minute supplemental oxygen via nasal cannula. Progression of COVID-19 infection was determined to be the cause of hypoxemia. He needed supplemental oxygen for two days in total. Urine color started to normalize after two days after the start of the treatment. The liver enzymes and CK levels gradually decreased. After seven days, hemin treatment was discontinued. On the 10th day, liver enzymes and CK levels were within the normal range and the patient had no symptoms.

**Table 1:** Laboratory workup on admission of the patient BUN: Blood urea nitrogen; AST: Aspartate aminotransferase; ALT: Alanine transaminase; GGT: Gamma-glutamyl transferase; ALP: Alkaline phosphatase; LDH: Lactate dehydrogenase; CRP: C-reactive protein; INR: International normalised ratio; WBC: White blood cells; NEUT: Neutrophil; RBC: Red blood cells; HGB: Hemoglobin; HCT: Hematocrit; MCV: Mean corpuscular volume; MCHC: Mean corpuscular hemoglobin concentration; RDW: Red cell distribution width; PLT: Platelet; MPV: Mean platelet volume; LYMPH: Lymphocyte; MONO: Monocyte.

Laboratory Workup On Admission											
Parameter	Patient	Reference Interval	Parameter	Patient	Reference Interval	Parameter	Patient	Reference Interval	Parameter (Urinalysis)	Patient	Reference Interval
BUN (mg/dL)	11.5	8.0 - 22.0	Total Bilirubin (mg/dL)	0.5	0.2 - 1.2	WBC (10^3^/μL)	15.6	4.3 - 10.3	pH	6	4.8 - 7.4
Creatinine (mg/dL)	0.87	0.7 - 1.4	Direct Bilirubin (mg/dL)	0.16	0 - 0.3	NEUT (10^3^/μL)	13.7	2.8 - 11.0	Urine Specific Gravity	1014	1.005 - 1.020
Urea (mg/dL)	24.61	0 - 50	CRP (mg/L)	31.26	0 - 5	RBC (10^6^/μL)	5.48	4.4 - 5.7	Ketone	Negative	
Uric Acid (mg/dL)	5.2	2.5 - 7.5	Fibrinogen	234.2	180 - 350	HGB (g/dL)	15	13.6 - 17.2	Nitrite	Negative	
AST (IU/L)	223.4	5 - 43	D-Dimer (μg/L)	344	0 - 550	HCT (%)	44.6	41 - 53	Protein	2+	
ALT (IU/L)	62.2	5 - 45	INR	1.05	0.85 - 1.2	MCV (fL)	81.4	80.7 - 95.5	Glucose	Negative	
GGT (IU/L)	26	5 - 85	Sodium (mmol/L)	136	135 - 146	MCHC (g/dL)	33.6	33 - 36	Bilirubin	Negative	
ALP (IU/L)	98	40 - 130	Potassium (mmol/L)	3.82	3.5 - 5.1	RDW (%)	13.6	11 - 15	Urobilinogen	Negative	
LDH (IU/L)	358	135 - 250	Chloride (mmol/L)	97	95 - 107	PLT (10^3^/μL)	184	155 - 375	Erythrocyte	1+	
Amylase (IU/L)	85	25 - 110	Calcium (mg/dL)	8.98	8.5 - 10.5	MPV (fL)	10	7 - 11.5	Erythrocyte Cell	10	
Lipase (IU/L)	21.3	0 - 60	Phosphate (mg/dL)	3.64	2.7 - 4.5	LYMPH (10^3^/μL)	0.7	1.2 - 3.6	Leukocyte Cell	0	
Albumin (g/dL)	4.96	3.2 - 5.5	Magnesium (mmol/L)	0.76	0.7 - 1	MONO (10^3^/μL)	1.1	0 - 0.8	Epithelial Cell	1	

## Discussion

In our case, the patient went two years without having an attack and there was no evident cause other than COVID-19 infection that might have triggered the attack. It is plausible that COVID-19 and AIP are two synergistic conditions amplifying one another given the interference of COVID-19 in the heme synthesis pathway and the severity of the AIP attack [4,5]. This result may also provide a basis for a combined approach to treat both conditions. A good example of this is hemin therapy [7]. Hemin works by inhibiting ALAS and inducing HO-1, which reduces systemic inflammation [6].

The elevated liver enzyme pattern was compatible with rhabdomyolysis even though an acute hepatitis due to the toxic effects of hemin on the liver could not be ruled out. We did not discontinue hemin therapy, since we believed that hemin was the most effective treatment available.

It was noteworthy that at the onset of the AIP attack, the patient had no respiratory symptoms. He developed hypoxemia and needed nasal intermittent positive pressure ventilation (NIPPV) while the symptoms primarily related to AIP (abdominal pain, nausea, urine color) were on the course of regression. From this, it might be inferred that COVID-19 could potentially affect heme metabolism even during the asymptomatic phase. This is likely due to the direct viral effects of SARS-CoV-2 rather than cytokine storm created at later stages. Given the fact that infections and systemic inflammation could also trigger and potentialize the effect of an AIP attack, cytokine storm could have aggravated the attack even further unless the treatment had been started during the asymptomatic phase.

The patient being unvaccinated against COVID-19 has likely contributed to the severity of the disease.

## Conclusions

COVID-19 is an infectious disease accompanied by systemic inflammation, particularly in people with underlying medical conditions. Cytokine storm syndrome, associated with significant systemic inflammation, is one of the leading factors behind the severity of COVID-19 infections. It has also been shown that COVID-19 interferes with heme metabolism. Given the fact that AIP is a disorder due to the impairment in the heme synthesis pathway and might be triggered by systemic inflammation, COVID-19 creates a basis for a possible AIP attack. Due to the mutual interaction of both conditions, the attack could be more severe than anticipated and we highly recommend monitoring patients with a history of porphyria closely during COVID-19 infection.
